# Immunoglobulins, Mucosal Immunity and Vaccination in Teleost Fish

**DOI:** 10.3389/fimmu.2020.567941

**Published:** 2020-10-02

**Authors:** Yongyao Yu, Qingchao Wang, Zhenyu Huang, Liguo Ding, Zhen Xu

**Affiliations:** ^1^Department of Aquatic Animal Medicine, College of Fisheries, Huazhong Agricultural University, Wuhan, China; ^2^Laboratory for Marine Biology and Biotechnology, Qingdao National Laboratory for Marine Science and Technology, Qingdao, China; ^3^Key Laboratory of Marine Biotechnology of Fujian Province, Institute of Oceanology, Fujian Agriculture and Forestry University, Fuzhou, China

**Keywords:** evolution, immunoglobulins, mucosal immunity, mucosa-associated lymphoid tissues, teleost

## Abstract

Due to direct contact with aquatic environment, mucosal surfaces of teleost fish are continuously exposed to a vast number of pathogens and also inhabited by high densities of commensal microbiota. The B cells and immunoglobulins within the teleost mucosa-associated lymphoid tissues (MALTs) play key roles in local mucosal adaptive immune responses. So far, three Ig isotypes (i.e., IgM, IgD, and IgT/Z) have been identified from the genomic sequences of different teleost fish species. Moreover, teleost Igs have been reported to elicit mammalian-like mucosal immune response in six MALTs: gut-associated lymphoid tissue (GALT), skin-associated lymphoid tissue (SALT), gill-associated lymphoid tissue (GIALT), nasal-associated lymphoid tissue (NALT), and the recently discovered buccal and pharyngeal MALTs. Critically, analogous to mammalian IgA, teleost IgT represents the most ancient Ab class specialized in mucosal immunity and plays indispensable roles in the clearance of mucosal pathogens and the maintenance of microbiota homeostasis. Given these, this review summarizes the current findings on teleost Igs, MALTs, and their immune responses to pathogenic infection, vaccination and commensal microbiota, with the purpose of facilitating future evaluation and rational design of fish vaccines.

## Introduction

Aquatic environments, which are inhabited by teleost fish, provide more nutrients to microbes than land ecosystems and therefore are more conducive to bacterial growth (1). Therefore, potential pathogens mostly enter the bodies of fish across their mucosal epithelial barriers including the gills, gastrointestinal system or skin lesions (2). Unlike invertebrates, teleost fish has evolved both innate and adaptive immunity to protect themselves against pathogens residing in their aquatic environment. Similar to mammals, teleost fish also produce B- and T-cells, which constitute the first adaptive immunity mechanisms in all bony vertebrates. Particularly, B-cells and immunoglobulins (Igs) in mucosal-associated lymphoid tissues (MALTs) are thought to mediate mucosal homeostasis, given that secretory Igs (sIgs; i.e., antibodies) are known to neutralize pathogens or promote their elimination in the mucosa, thereby preventing further infection (3). Recent studies have demonstrated that B-cells within the mucosal tissues, including the gills, buccal mucosa (BM), pharyngeal mucosa (PM), and olfactory organ, exhibit potent local proliferation (4–6). Additionally, the large commensal microbiota populations that colonize fish mucosal surfaces can be recognized by Igs (7). So far, three main Ig isotypes have been identified in teleosts including IgM, IgD, and IgT/Z (8, 9), of which IgT/Z is thought to be specialized in mucosal immunity. The predominant roles of IgT/Z antibodies and IgT^+^ B-cells have been elucidated in six different teleost MALTs, including GALT (10), SALT (11), GIALT (4), NALT (12), and buccal (5) and pharyngeal MALT (6).

The present review aims to summarize the regulatory functions of Igs in teleost mucosal immunity. First, we will review the basic current information on the three known teleost Igs at both the gene and protein levels. Afterward, we will describe the six different MALTs in teleosts. We will then discuss teleost Ig responses to pathogens and microbiota in mucosal surfaces and finally teleost Ig responses to immunization. Altogether, the information compiled herein will provide insights into the fundamental mechanisms of Igs in fish mucosal tissues and aid in the development of novel teleost mucosal vaccines.

## Immunoglobulins

Igs are highly specialized recognition glycoproteins that can recognize a great variety of antigens from bacteria, viruses, and other disease-causing organisms and recruit other cells and molecules to destroy these pathogens (13). Igs are composed of two heavy (H) chains and two light (L) chains, with the exception of certain antibodies in camelids and nurse shark that lack L chains (14). The constant region within the heavy chain determines the effector function of a specific antibody, which in teleosts includes Cμ, Cδ, and Cτ/Cζ, encoding IgM, IgD, and IgT/IgZ, respectively (8, 9). Four L chain types have been identified in bony fish, of which Igκ and Igσ are found in most teleost species, Igλ has been lost in most teleost lineages during species divergence (cod, catfish, and rainbow trout are notable exceptions), and Igσ-2 was also recently identified in the coelacanth (15).

In tetrapods, naïve B-cells tend to produce IgM before any other isotype, but the germinal centers of secondary lymphoid tissues can induce B-cells to express a different antibody isotype *via* class switching when activated by antigens (16). However, class switching does not exist in any fish species including teleosts and cartilaginous fish, as there are no distinct switch regions in the gene locus (17) despite the identification of an activation-induced cytidine deaminase (AID) (18). Two forms of Igs are reported in both teleosts and tetrapods, a membrane-bound form of Igs, also known as the B-cell receptor (BCR), which serves as the cell’s antigen receptor, and the secreted form of Igs, which is produced by terminally differentiated B-cells, plasmablasts, and plasma cells, and functions as the main effector of B-cells in adaptive immunity (19). Here, we mainly focus on the secreted teleost forms IgM, IgD, and IgT/Z.

### IgM

IgM is nearly universal in all jawed vertebrates, with only the exception of the African coelacanth that carries exclusively IgW H chain loci (20). In teleosts harbouring IgZ/T, the Cµ domain is not the first constant region after the VDJ domain, and additional Dτ-Jτ-Cτ/ζ domains are located before the Dµ/δ-Jµ/δ-Cµ-Cδ domains in the gene locus of the teleost IgH chain (8, 9). During V(D)J rearrangement in teleosts, the upstream VH could merge with the aforementioned Dµ/δ-Jµ/δ-Cµ-Cδ domains *via* Dτ-Jτ-Cτ/ζ domain deletion, which results in IgM synthesis.

Tetrameric IgM is widely accepted as the prevalent serum Ig type in most teleosts (13). Teleost IgM lacks the J chain, and its monomers are mainly associated by covalent (disulfide) bonds. Increased disulfide polymerization has been reported to correlate with a greater affinity of trout IgM to antigens, and this trout IgM was also reported to remain active for longer periods (21). Teleost serum IgM concentration may vary (0.6–16 mg/ml) depending on water temperature and quality, as well as fish species, size, stress, stimulation, and immunization (22). During parasitic infection, both the total serum IgM concentration and parasite-specific IgM binding capacity of trout were significantly increased, in addition to an increased IgM^+^ B-cell proliferation in head kidney (5, 6), which demonstrated the regulatory functions of IgM in teleost systemic immunity. IgM has also been shown to occur as a tetramer in the different mucus types of rainbow trout at different concentrations, including gut mucus (~0.075mg/ml), skin mucus (~0.0046 mg/ml), gill mucus (~0.02mg/ml), pharyngeal mucus (~0.072mg/ml), and nasal mucus (~0.28mg/ml), all of which exhibited lower IgM concentrations than serum (~2.5mg/ml). Nonetheless, IgM still comprises the largest fraction among all three Igs in all teleost mucus, and parasite-specific IgM binding in pharyngeal mucus has been shown to increase after parasitic infection. IgM has also been found to coat bacteria in different mucus types, albeit at a lower rate than IgT (4–7, 11). Moreover, IgM expression exhibits some general features in teleosts. For instance, cytoplasmic IgM is normally expressed earlier than surface IgM, and Ig-producing cells appear in different tissues in the following order: head kidney, spleen, and finally MALT (23). In zebrafish, ontogenic IgM expression patterns occur in the following order: surface Ig transcript [7 days post-fertilization (dpf)], IgH chain transcript in the pancreas (10 dpf), sIg transcript (13 dpf), IgH chain transcript in the head kidney (19 dpf), and detectable humoral Ig (28 dpf) (8, 24).

### IgD

Compared to the limited (i.e., normally two or three) Cδ domains in mammals, the number of Cδ domains varies widely in different fish species (25). Unlike eutherian δ chains, in which two domains are connected by a hinge, the higher number of Cδ domains in teleosts may provide a wider variety of structural options to synthesize more flexible H chain products. Moreover, δ in bony fish was found to be uniquely formed by splicing Cµ1 between rearranged VDJ and Cδ1, producing a chimeric H chain sequence (26). Notably, this inclusion of Cµ1 has been observed in almost every teleost Igδ transcript (27). In channel catfish, membrane bound and secreted IgD is transcribed from two different IgH genes. Interestingly, the secreted delta form is encoded by an IgH transcript lacking a V-region (25). A germline-recombined VDJ is present in the second gene, but the signal sequence is directly spliced to Cδ1 in the mRNA, indicating that the secreted δ form may not perform antigen recognition functions (17).

In teleosts, IgD was found to be to co-expressed with IgM in rainbow trout B-cells (4); however, an IgM^-^/IgD^+^ B-cell population was also identified in channel catfish (28) and a European trout line (29). In channel catfish, IgM^-^/IgD^+^ B-cells isolated from peripheral blood lymphocytes (PBLs) likely expand in response to certain pathogens, functioning as pattern recognition molecules. And a recent intriguing study in the trout identified a preponderant IgT^-^ B cells, i.e. IgD^+^ IgM^-^ cells, to secrete IgDs which were reactive to the commensal microbiota in both gut and gills but not skin (29). However, the immunoprotective role of IgD in teleosts remains largely unknown. Although some reports have hypothesized the role of IgD in teleost gills (26), this antibody is known to be uninvolved in specific immunity in the gills and PM of rainbow trout during parasitic infection (4, 6). Additionally, teleost sIgD can coat a subset of commensal microbiota in mucosal tissues, including the gut mucosa, gill mucosa, BM, and PM. Nonetheless, mucosal bacteria coating by sIgD occurs significantly less than sIgT coating (4–6). These results suggest that teleost sIgD may also be involved in mucosal homeostasis.

### IgT

IgT acts as a mucosal-associated Ig in bony fish, similar to IgA in mammals and IgX in frogs (10). Besides the salmonids fish (8, 9, 30, 31), IgT subclasses are also reported in other teleosts such as stickleback (*Gasterosteus aculeatus*) (32, 33) and carp (*Cyprinus carpio*). Differential immune responses have been observed which may vary depending on both species and the route of pathogens/vaccination used in the study. Similar results have been observed for other ig isotypes (34). In teleost B cells, the IgH V domain is known to rearrange either to Dτ-Jτ-Cτ/ζ to encode a τ/ζ chain or to Dμ/δ-Jμ/δ-Cμ-Cδ to encode μ and δ chains (19). Similar to IgD, the number of τ/ζ domains varied in different teleost species. For instance, four domains have been identified in most analyzed species (35); three domains in stickleback (33) and Antarctic fish (36); and two domains in fugu (37). Two separate IgZ loci encoding IgZ1 and IgZ2 have been identified in zebrafish (38) and common carp (39). IgZ1 represents a typical teleost IgHτ/ζ feature, whereas the Cμ 1 and Cζ 4 domains constitute a chimeric transcript of IgZ2. Moreover, very recent study has reported that multiple independent rounds of duplication and deletion of the teleost-specific antibody class IGHZ in the cyprinodontiform lineage, demonstrating the extreme volatility of IGH evolution (40).

In rainbow trout serum, IgT mainly occurs in monomeric form and is eluted at ~180 kDa, as determined from standard curve analysis. Within the multiple types of mucus, a great portion of IgT is present in polymeric form, whereas a small portion of IgT is still present in monomeric form during elution. Unlike the tetrameric IgM which is associated by covalent (i.e., disulfide) bonds, gut polymeric IgT is composed of non-covalent associated monomeric subunits. The concentration of IgT in the serum of rainbow trout is 4–10 mg/ml, whereas it is ~7 μg/ml, ~0.31 μg/ml, ~1.55μ g/ml, ~1.7 μg/ml, and ~3.46 μg/ml in the gut mucus, skin mucus, gill mucus, nasal mucus, and pharyngeal mucus, respectively. It is important to note that teleost mucus possesses a much higher ratio of IgT/IgM than serum. Moreover, mucosal microbiota is mainly coated with IgT in the gut, skin, gills, and olfactory organ (4–7, 10–12). Among the different mucosal tissues, it was also observed that, compared to the gills and gut, skin mucosa showed lower IgT concentrations, lower IgT titers, and a lower percentage of coated microbiota which were correlated with lower percentages of IgT^+^ B-cells in the skin of the same fish (4, 10). The abundance of IgT^+^ B-cells within a specific mucosal surface may be driven by its commensal microbiota, which then modulates humoral immune response potency (7).

## Mucosa-Associated Lymphoid Tissue (MALT)

**Figure 1:**
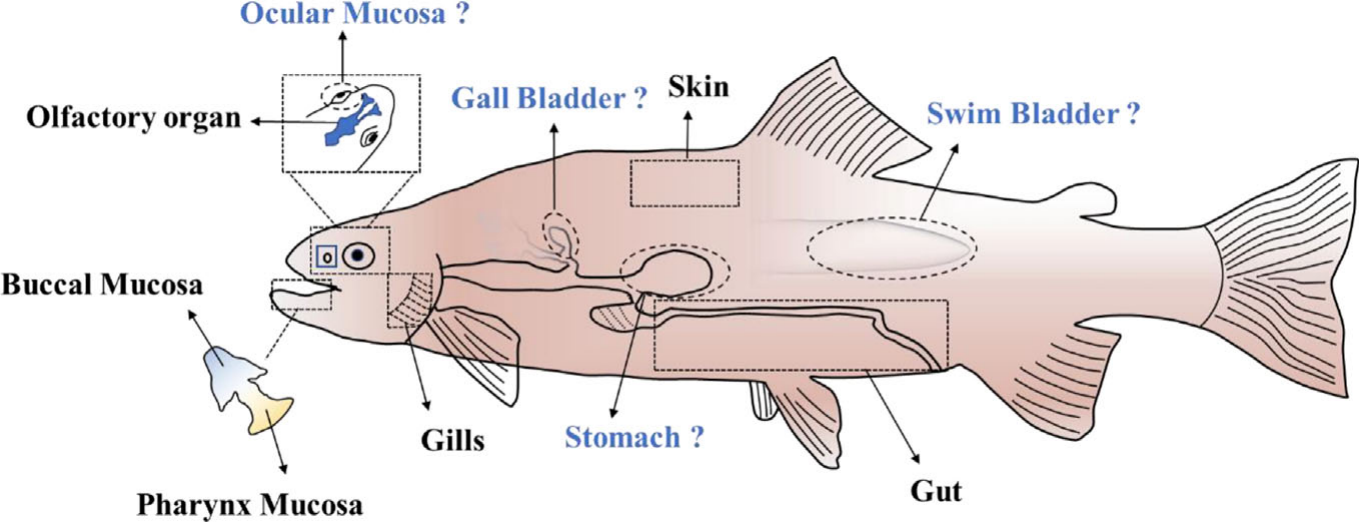
The MALTs in teleost. “?” indicates the potential MALTs in teleost fish which remain to be clearly delineated.

So far, six different MALTs (classified based on their localization in the body) have been identified in teleost fish (**Figure 1**), which contain diffuse MALTs (D-MALTs) but lack the organized MALTs (O-MALTs) such as Peyer’s patches and tonsils that are found in mammals. These organized structures are the inductive sites for the selection of high-affinity B-cells and are believed to facilitate antibody response maturation (41). Teleost MALTs harbor abundant myeloid and diffuse lymphoid cells, which can work together to initiate both innate and adaptive immunity to maintain mucosa homeostasis.

### Gut-Associated Lymphoid Tissue (GALT)

The digestive tract is directly connected with the external environment and could be a main portal of pathogen entry in both mammals and teleost fish, among which gut is the largest part (42). As a crucial component of the mucosal immune system in teleost fish, GALT constitutes a local immune response environment for protection against pathogens. Although the gut structure varies in different teleost species, it can be generally divided into three main segments (43). The enterocytes of the first segment act as absorptive cells for dietary protein uptake. The second segment is involved in macromolecule and enterocyte uptake. The third segment is thought to have osmoregulatory functions. In addition to the digestive function of the teleost gut, studies have shown that the posterior segment of the teleost intestine contains several immune cell types (10, 17, 44), which suggests that this section is involved in immune responses against pathogen invasion. Research on gut-associated immune responses in fish have been primarily investigated in the study of T-cell function (45); Moreover, three B-cell populations (IgM^+^IgD^+^, IgM^-^IgD^+^, and IgT^+^ B-cells) in the intestine of rainbow trout have been described (10, 29). Particularly, IgM^+^ and IgT^+^ B-cells have mostly been located in the lamina propria (LP), whereas some studies have shown that IgT^+^ B-cells in carp and sea bass are primarily localized in intraepithelial lymphocytes (IELs) (10). This suggests that due to the privileged population of IgT^+^ B-cells, differential immune responses can be effortlessly generated upon infection or vaccination.

### Gill-Associated Lymphoid Tissue (GIALT)

Unlike terrestrial animals that acquire oxygen from the air and possess invaginated breathing structures (i.e., lungs), aquatic organisms possess evaginated gas-exchange structures. In the case of teleost fish, four pairs of gill arches consisting of many gill filaments constitute a highly efficient way to increase contact surface to acquire oxygen from water. However, in addition to the respiratory function of the gills, they also possess osmoregulation, pH balance regulation, ammonia excretion, hormone regulation, and detoxification functions (46). Notably, because of their direct exposure to water, teleost gills are continuously challenged with environmental pollutants/toxins and pathogens, both of which trigger an immune response in the teleost GIALT (47). Moreover, several studies have demonstrated that numerous innate and adaptive immune molecules or cells involved in immune-related pathways are present in teleost gills, such as Igs and antibody-secreting cells (4, 48). It has been noted that the IgT^+^ B-cell locus on the epithelial layer plays an essential role in the defense against pathogens (4). Moreover, unlike IgM^+^IgD^+^ B-cells, IgM^-^IgD^+^ B-cell populations are also present in the gills of rainbow trout (29), but the function of these cells remains to be clearly characterized.

### Skin-Associated Lymphoid Tissue (SALT)

The skin of teleost fish also acts as the first line of defense against invading pathogens and it contains SALT, which can elicit gut-like immune responses against pathogen infection/antigen stimulation (11, 49). Compared with GALT, SALT might play a key role in defense against pathogens during the earlier developmental period of the teleost fish. Recently, a model of a germ-free zebrafish embryo challenged with osmotic stress showed that the embryo skin acts as the first organ to protect the organism from bacterial infection during the early developmental period when GALT was not yet functional (50). In general, all vertebrates possess two main skin layers: the epidermis and the dermis. However, unlike mammals, teleost skin is not keratinized and therefore their epithelial cells with abundant mucus-producing cells are in direct contact with the water medium (13, 51). Additionally, the skin surface of teleosts generates numerous molecules including lysozymes, complements, lectins, and Igs, all of which attach to the skin mucus to protect the host (52). Skin mucus in most fish contains numerous innate immune components, which can be produced continuously to prevent the entry of pathogens to the underlying tissues. Importantly, fewer IgT^+^ and IgM^+^ B-cells are found in teleost SALT than in GALT. However, teleost skin B cells are dominantly located in the epidermis, and almost none of them are found in the basal dermis layer (11).

### Nasal-Associated Lymphoid Tissue (NALT)

Olfaction is an essential ancient sensory system prevalent in all animals. Interestingly, the olfactory systems of teleost fish resemble those of land-based animals in terms of anatomical features (53). Given their distinctive environment, teleosts have developed different mechanisms to discriminate odors. Terrestrial vertebrates breathe and sense their environment through inhalation, whereas teleost fish actively draw water containing dissolved gases into their olfactory organs (54). Additionally, teleost olfactory organs are continually stimulated with pollutants/toxins or pathogens in the water (55). Therefore, teleost NALTs are similar to other teleost MALTs, containing diffuse lymphoid cells without organized structures, and IgT^+^-B cells play a crucial role in pathogenic invasion defense.

### Buccal Mucosa-Associated Lymphoid Tissue

The buccal cavity, located between the gastrointestinal and respiratory tracts, is a vital mucosal surface in vertebrates. In mammals, the BM contains numerous salivary glands to produce saliva, which subsequently translocates into the salivary layer (SL). Moreover, the buccal cavity (BC) is covered with a keratinized stratified epithelium associated with the gingiva, hard palate, and outer lips and a non-keratinized stratified epithelium in other areas (56). Conversely, the BC of teleost fish is lined with abundant mucus-secreting cells that replace the function of the salivary glands in mammals to produce the mucus to coat their buccal epithelium (57). Furthermore, only non-keratinized buccal epithelia have been observed in teleost fish, which exhibit similar characteristics to those of the mammals. Specifically, this epithelium consists of two main layers: the stratified squamous epithelium and the LP (58, 59). Given that aquatic environments are far more complex than air, teleost fish are subject to more stimuli. A recent study reported that fish BC also contains MALTs as in other mucosal tissues such as skin and has evolved an effective mucosal immune system to protect itself from parasite infection (5).

### Pharyngeal Mucosa-Associated Lymphoid Tissue

The pharynx is connected with the digestive and respiratory tracts in vertebrates (60). In mammals, the pharyngeal cavity (PC) contains a choana which is connected with the nasal cavity (NC). However, in teleost fish, the PC (which is located between the mouth and esophagus) is a separate compartment from the NC and does not contain a choana. The PC of teleosts is coated with mucosa, and contains the stratified squamous epithelium and the LP (61). Unlike the PC of mammals, which possesses both mucus-secreting cells and mucus glands to secrete mucus into the mucus layer (62), aquatic animals lack mucus glands and instead possess numerous mucus-secreting cells in the PM. Similar to the buccal MALT, IgT^+^ B-cells are chiefly located in the pharyngeal epithelium (PE), where their numbers increase significantly upon pathogenic invasion (6).

### The Potential Mucosa-Associated Lymphoid Tissues Which Might Present in Teleosts

In addition to the six MALTs mentioned above, four potential extra MALTs might be present in the teleosts. Ocular mucosa of the teleost fish is directly exposed to the water medium. As one of the mucosa-associated lymphoid tissues in mammals, ocular mucosa plays an important role in defense against the pathogen invasion. From an evolutionary perspective, ocular mucosa in teleost fish might also have a role in protecting the individual from environmental pathogens. The stomach is another component of the digestive tract that has a potential role as MALT. Recent research has shown that IgA plays an important role in maintaining homeostasis of the stomach mucosa in mammals (63). In contrast, whether IgT/IgZ in teleosts perform a similar role as IgA in stomach remains to be demonstrated. The gallbladder is also an accessory organ of the digestive tract, and it greatly influences bile inflow into the intestine and thereby the enterohepatic circulation of bile acids. Therefore, it is closely connected to the intestine and might defense against pathogen invasion together with the intestine. Finally, the swim bladder which connects with the esophagus *via* the pneumatic duct is a homologous structure to the tetrapod lung (64). Its primary function is to control whole-body density and buoyancy. Recently, swim bladder-associated microbiota in rainbow trout was investigated and the result showed that *Arthrobacter* and *Cellulosimicrobium* were the major genera located in the swim bladder mucosa (65). The cross-talk between resident bacteria and host, and whether a connection exists between the swim bladder and the gut microbiota, is a highly interesting hypothesis.

## Immunoglobulin Responses to Pathogens in Mucosal Surfaces

Fish are continuously exposed to an aquatic environment that contains abundant pathogens such as bacteria, viruses, and parasites, all of which can break through the immune barrier of the body. The mucosal immune system of fish is highly efficient and plays a critical role in resisting various pathogens, as previously reported in mammals. Many functions of mammalian IgA in mucosal secretions have been confirmed, such as preventing pathogens from adsorbing to the mucosal epithelium, mediating virus neutralization in infected cells, and promoting the death of pathogens by activating the alternative complement system pathway (66). To date, IgM, IgD, and IgT/IgZ have been identified in teleost fish and IgT/Z are the main immunoglobulin isotypes specialized in mucosal immunity (4, 7, 10).

### Immunoglobulin Responses to Pathogens in the Gut

The Igs in the teleost gut play an important role in resisting the invasion of potential pathogens through its epithelium (67). In 2010, Zhang et al. identified IgT as the most ancient known Ig specialized in mucosal immunity (10). In their report, more IgT^+^ B-cells were detected in the GALT of fish infected with *Ceratomyxa shasta* (a gut parasite) than that of control fish; however, the percentage of IgM^+^ B-cells was not higher than that of control fish. Importantly, the authors identified parasite-specific IgM titers in serum, whereas IgT-specific responses to the parasite were confined to the gut mucus, which demonstrated that IgT might play a major role in pathogen inhibition in the fish gut. The above results suggest that teleost IgT contributes exclusively to gut mucosal immune responses, which is similar to the role of mammalian IgA, whereas IgM plays a major role in systemic immunity. In mammals, the secondary lymphoid follicles in the gut (e.g., Peyer’s patches; PP) are responsible for the proliferation and enrichment of IgA^+^ B-cells (68). However, unlike mammals, teleosts lack PP, suggesting that the proliferation of IgT in the GALT of trout occurs through other pathways. In a later study on gilthead seabream (*Sparus aurata*), increased IgM expression in the posterior intestine was found in fish infected with *Enteromyxum leei*. Additionally, an increased number of IgM^+^ B-cells was also detected in the intestine of infected fish (69). Natalia et al. described that after rainbow trout were orally infected with infectious pancreatic necrosis virus (IPNV), the transcript levels of many immune-related genes significantly changed and the gene expressions of IgM and IgT were higher in the pyloric ceca than that in kidneys (70). So far, reports on Ig responses in intestinal mucosa after viral infection are still scarce. To further clarify the critical role of Igs against pathogens in the intestine of fish, additional bacterial and viral studies should be conducted.

### Immunoglobulin Responses to Pathogens in Gills

Previous studies have demonstrated that gill tissues contain abundant immune cells, which are regulated by several immune genes and pathways (71–73). A later study examined the specific Ig responses to pathogens in fish gills (4). In this study, *Ichthyophthirius multifiliis* (Ich) trophonts in the gills were overwhelmingly coated with IgT and slightly coated with IgM at 25 days post-infection (dpi), whereas parasites coated with IgD could not be detected. Moreover, a significantly increased number of IgT^+^ B-cells in the gills could be detected in infected and survivor fish compared to control fish. In contrast, IgM^+^ B-cell numbers did not show obvious changes. Additionally, at the protein level, IgT protein concentration also increased significantly in the gill mucus of infected and survivor fish, whereas the IgM and IgD protein concentrations remained unchanged. Conversely, the concentration of IgM in serum showed a significant increase (unlike IgT). Moreover, high titers of parasite-specific IgT and parasite-specific IgM were detected in gill mucus and serum, respectively. Similar results were obtained when detecting the titers of bacteria-specific IgT/IgM in gill mucus of fish infected with *Flavobacterium columnare*. More details about the Ig responses in trout gills infected with *F. columnare* were provided in that study (74). Additionally, significant upregulation of genes encoding IgM and IgT was induced by immunization and challenge with Ich in teleost fish gills (75). Moreover, upon bath immunization, the expression of the IgT gene in the gills could be induced by treating *Epinephelus coioides* with a nervous necrosis virus vaccine (34). To elucidate the roles of Igs against different pathogens in fish gills, further studies should be conducted to provide insights into the gill responses in virus-infected fish.

### Immunoglobulin Responses to Pathogens in Skin

Previous studies have found that IgT protein concentration significantly increases in skin mucus upon Ich infection and coats the surface of the invasive parasite (Ich), suggesting the critical function of IgT in skin infection resistance (11). In this study, rainbow trout infected with Ich presented a significant increase in IgT^+^ B-cells in the skin at 3 months post-infection. However, IgM^+^ B-cell numbers remained largely unchanged in infected groups. Additionally, all parasites localized within the skin epidermis were largely covered with anti-IgT antibodies. At the protein level, the IgT protein concentration in the skin mucus of infected and survivor fish was significantly higher, whereas IgM levels remained unchanged. Similar to the results in fish gut, the presence of parasite-specific Igs was discovered in skin mucus and serum. Moreover, when fish treated with mucosal vaccination were bath-challenged with pathogens, the IgM titers in serum and IgT responses in skin mucus were strongly induced (34). Furthermore, in Ich-infected fish, a significant upregulation of IgT, IgM, and IgD levels was observed in the skin at 24 h post-infection (76).

### Immunoglobulin Responses to Pathogens in the Olfactory Organ

The nasal mucosa is known to inhibit and neutralize pathogens and participates in both local innate and adaptive immune responses (12, 77). In Ich-infected trout, parasites in the olfactory organ were mostly coated with IgT, slightly coated with IgM, and hardly coated with IgD. The nasal epithelium also produced notable IgT^+^ B-cell accumulation when the fish survived the parasite. Moreover, in agreement with the results that nasal IgT^+^ B-cells increased in infected fish, the IgT protein concentration in the nasal mucosa of these fish was also noticeably increased, whereas IgM and IgD protein concentrations did not show any significant changes. Contrary to the results in the nasal mucosa, the IgM concentration in the serum of infected fish significantly increased. A later study determined that parasite-specific IgT was secreted in the nasal mucosa in response to Ich infection. Additionally, Magadan et al. reported that enteric red mouth (ERM) intranasal administration resulted in a significant perturbation of the IgM (IgH μ) repertoire in trout spleen, and the IgT repertoire showed a lower diversity and higher relative IGHV2 usage compared with the controls. However, no ERM-specific IgT was detected in serum after intranasal immunization, suggesting a lower capacity to activate plasmatic B-cell differentiation or to stimulate their migration to the head kidney (78). Interestingly, Sepahi et al. reported that the percentage of IgT^+^ B cells markedly decreased in the olfactory organ of IHNV-treated trout, whereas IgM^+^ B cells did not change significantly (79). Therefore, to further our knowledge of Ig responses to pathogens in fish nasal mucosa, immune responses induced by bacterial and viral infections in the nasal mucosa should be further studied.

### Immunoglobulin Responses to Pathogens in BC

In tetrapod species, sIgA plays a critical role in adaptive immune responses to prevent the invasion of oral pathogens as a pivotal humoral component (80). Recently, studies of Ig responses to pathogens in the BM of fish have also been conducted (5). The highest innate immune response intensity in fish was detected at days 14 and 28 post-Ich infection, including the expression of IgM, IgT, and IgD heavy chain genes. Moreover, similar to other fish MALTs, the accumulation of IgT^+^ B-cells could also be observed in the buccal epithelium of surviving fish and, similar to previous studies, the number of IgM^+^ B-cells did not change significantly. In line with the increased number of IgT^+^ B-cells in the buccal epithelium, a significantly increased IgT protein concentration was detected in the buccal mucus of infected and surviving fish. In contrast, the concentrations of IgM and IgD did not significantly change in the buccal mucus of the infected group. However, the concentration of IgM increased significantly in the serum of infected fish. Moreover, compared to the Igs concentration of control fish, the titers of parasite-specific IgT were much higher in the buccal mucus of infected fish, as demonstrated by a pull-down assay, whereas the titers of parasite-specific IgM increased mainly in the serum. However, no parasite-specific IgD was detected in either the buccal mucus or the serum.

### Immunoglobulin Responses to Pathogens in PC

B cells and Ig are present in the PM of teleost fish (6). In fish challenged with Ich for 28 days, IgT^+^ B-cells increased dramatically in the PE and were found to secrete IgT. In contrast, the number of IgM^+^ B-cells in the pharynx of the infected group remained at the same level as that of the controls. Moreover, the IgT concentration in the pharyngeal mucus was also obviously upregulated, whereas the protein concentrations of IgM and IgD in the pharyngeal mucus remained unchanged after Ich infection. However, in the serum, higher IgM and IgT but not IgD concentrations were detected in infected fish. Upon further pull-down experiments, the titers of parasite-specific IgT and IgM were tested in both pharyngeal mucus and serum. The titers of parasite-specific IgT were higher in pharyngeal mucus, whereas the titers of parasite-specific IgM were mainly detected in serum.

## Immunoglobulin Responses to Microbiota at Mucosal Sites

**Figure 2:**
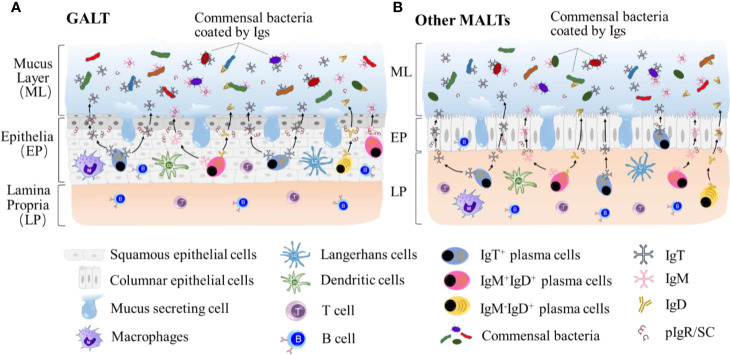
Teleost IgT, IgM and IgD are produced by IgT^+^, IgM^+^IgD^+^ and IgM^-^IgD^+^ B-cells mainly located in lamina propria in GALT **(A)** and epithelial layer other MALTs, such as GIALT, SALT, NALT, PM and buccal MALT **(B)**. Igs are transported from the epithelium into the mucus layer by the teleost pIgR to maintain the homestasis of microbiota at mucosal sites. The symbiotic bacteria in the mucus is predominantly coated by sIgT.

Commensal microbes are very important to overall fish healthy, some of which have been demonstrated to be involved in the protection of fish against infectious agents (81). Fish MALTs have developed a complex immune system to interact with microbiota at mucosal sites, and the relationship between them provides mutual benefits at homeostatic conditions. Maintaining the mucosal microbial homeostasis is critical for the maintenance of the mucosa and ultimately whole-body health. Thus, understanding the mechanisms by which teleosts MALTs are involved is of critical importance. In the teleost experiments described above, three Igs have been characterized in their mucosal secretions (IgM, IgD, IgT), where IgT is the most ancient antibody class specialized in mucosal immunity (10). Interestingly, it has been found that the interaction between teleost Igs and microbiota *via* coating (**Figure 2**) is similar to that in mammals (80), whereas the percentage of Ig-coated bacteria varies at individual mucosa sites. This demonstrates that microbiota control at mucosal sites has emerged early in the evolution of vertebrates and is conserved. Here, we review some current studies on teleost fish Ig immune responses to microbiota.

### Immunoglobulin Responses to Microbiota in the Gut

The intestinal mucosal surface interfaces of vertebrates, including teleost fish, are colonized with large and complex microbial populations. Although microbiota composition in teleost intestines is influenced by the aquatic environment or the diet, microbial content is generally maintained at approximately 10^7^–10^8^ bacteria per gram, among which aerobic and facultative anaerobic organisms are dominant (17). In some cases, the teleost-gut-associated microbiota is also essential for body health, as zebrafish devoid of microbiota showed impaired innate immune functions when compared to normal fish (82). Alternatively, under the effects of a stressor, symbiotic bacteria can also turn into pathogenic bacteria, thereby causing disease or even death (83). Therefore, the role of GALT in the control of homeostasis of commensal microorganisms is crucial to support fish health (84, 85).

In mammals, the involvement of IgA in the coating of both beneficial bacteria and opportunists promote the preservation of microbiota homeostasis on mucosal surfaces (84, 86). Given that IgT appears to be a dedicated mucosal Ig in teleosts, IgT not only plays a key role in pathogen response but is also implicated in intestinal homeostasis by recognizing and coating bacterial microbiota (10). In a previous study, almost half of the microbiota present on the intestinal surface were found to be coated with IgT, whereas IgM and IgD could only bind to a small and similar percentage of bacteria (10, 29). As the Ig-bacteria interaction in teleosts is currently unclear, further studies are needed to shed light on the function og IgT, IgM and IgD with respect to bacterial coating and gut homeostasis.

### Immunoglobulin Responses to Microbiota in Gills

Similar to the function of sIgs in mammal lungs (87), Igs in gill mucus can maintain immune homeostasis by limiting microbial antigen access to the fish body, thereby keeping the epithelial barrier’s integrity and shaping the composition of the symbiotic microbiota (4, 7, 74). One study demonstrated a large population of bacteria in the gill mucosa was predominately coated by sIgT and to a much lesser degree by IgM and IgD, indicating the conserved role of a specialized mucosal Ig in vertebrates that recognize commensal bacteria on the gas-exchange surface (4). Upon IgT depletion, it was clearly observed that the percentage of IgT-coated microbiota decreased significantly and recovered when IgT was re-produced in the fish gill mucosa. Moreover, a set of microbes translocated from the mucus layer across the gill epithelium and invaded the systemic circulation in sIgT-depleted fish. IgT-seq demonstrated that IgT coats a broad but well-defined range of bacteria with both beneficial and pathogenic characteristics and microbiota composition changed significantly, which demonstrated the loss of potentially beneficial bacteria that produce short-chain fatty acids (SCFAs) and pathobiont expansion after IgT depletion. Interestingly, when the level of IgT was restored to its original state, the balance of microbiota was reestablished in the gill surfaces (7). Thus, these observations strongly imply that teleost IgT is essential to maintain microbiota homeostasis in the gills.

Additionally, *Flectobacillus major*, a bacterial species that dominates trout gill, can induce specific IgT secretion in the gills of healthy rainbow trout (55). It is assumed that *F. major* modulates Igs and then regulates the symbiotic homeostasis in the gill surface. However, its specific mechanisms still need to be determined. Thus, future studies should investigate not only the functional mechanisms by which Igs regulate the commensal microbiota and the role of IgT/IgZ in targeting specific bacteria in teleost fish gills, but also the mechanisms by which certain components of the commensal microbiota affects mucosal and systemic immunity.

### Immunoglobulin Responses to Microbiota in Skin

The skin of teleosts contains a layer of live cells covered by a rich mucous layer with high densities of bacterial microbiota (88, 89). Under normal circumstances, the skin mucosal immune system of teleosts is in a dynamic equilibrium with symbiotic microorganisms, and skin homeostasis is critical to preserve teleost health in vertebrates. However, once the original balance is disturbed by external factors such as parasitic infection, bacterial community composition changes significantly, thereby leading to pathobiont proliferation and secondary infection (76). Thus, an understanding of the mechanism by which microbial homeostasis is maintained is necessary for the study of skin immunity in teleosts. So far, it has been demonstrated that teleost sIgs, especially sIgT, play a significant role in the control of skin symbiotic flora homeostasis. In a study on rainbow trout, IgT was found to be bound to a large fraction of skin bacteria (∼38%), whereas only a small proportion (∼12%) was coated with IgM (11), which is consistent with the mammalian sIgA coating of skin bacterial microbiota (90). The targeting of skin microbes by mammalian sIgA plays a key role in immune exclusion (90); however, mucosal Ig can promote the colonization of certain bacteria on the skin surface (91). Therefore, considering that teleost skin is a unique mucosal surface that elicits gut-like immune responses, future research should determine whether teleost sIgT is involved in immune exclusion or assists with bacterial colonization.

### Immunoglobulin Responses to Microbiota in the Olfactory Organ

The olfactory organs of vertebrates are extraordinary chemosensory structures that harbor a variety of symbiotic microorganisms in the NC (12). In mammals, nasal symbiotic bacteria play an important role in controlling immunological development *via* different mechanisms including inhibiting pathogen colonization on the mucosal surface and stimulating the host immune system (92). In contrast, the function of microbiota in the NC of teleost fish has not been determined in the olfactory organ of teleost fish; however, the bacterial community in NC of rainbow trout is dominated by *Proteobacteria*, *Actinobacteria*, *Bacteroidetes*, and *Firmicutes* (93), among which *Proteobacteria* and *Firmicutes* may be the most conserved taxa in the olfactory organ of vertebrates (55).

The nasal bacteria of teleosts are also anchored by sIgs, similar to those in the gul, gill and skin, to the mucus layer through a mechanism known as immune exclusion (12, 55). However, sIgT and sIgM in the olfactory organ coat equal proportions of microbiota in contrast to skin, gut and gills where IgT is specialized in coating, and IgM coats significantly lower numbers of bacteria (10, 11). This may be because the isotypes of Ig coating mucosal bacteria on MALTs are different due to the diversity of local microbiota (93). Alternatively, as the percentage of nasal commensal bacteria coated with both IgM and IgT is much higher than that of other mucosal sites, it is possible that the extent of Ig binding-microbiota varies in trout depending on genetic lines or ages, and some of the bacterial taxa coated by sIgM are those bacteria found initially coated by sIgT. Thus, a future Ig-seq study of the species of microbiota coated by IgT/IgZ and IgM may confirm the aforementioned possibilities.

### Immunoglobulin Responses to Microbiota in BC

The BC is covered with a critical layer of mucus that is constantly exposed to microorganisms from air, water, and food in vertebrates. Similar to humans, *Proteobacteria* and *Actinobacteria* are dominant in the BC of teleosts (94, 95). However, the bacterial communities differed post-infection when challenged with hematopoietic necrosis virus (IHNV); that is, the abundance of *Pseudomonas* significantly decreased, while the abundance of *Clostridiales*, *Bacteroidales*, and *Escherichia-Shigella* increased, all of which are related to human intestinal diseases (96–98). As previously described for skin sites, pathogenic infection may break the homeostasis of microbiota in the BM, facilitate the colonization of opportunistic bacteria, and then cause secondary bacterial infections in the BC (95). Thus, the microbial homeostasis of the teleost BM is necessary for buccal health.

It is important to point out that the BMs of vertebrates have evolved an efficient immune system to maintain homeostasis (5). Moreover, the mucosal molecular responses of fish and mammals (i.e., sIgT versus sIgA) utilize different but functionally analogous strategies to initiate a response to microbiota in the BC (5). Due to its constant interaction with the water environment, the homeostasis of fish BM microbiota may be prone to frequent adverse effects. Among the three teleost Igs, sIgT is also the main Ig class that binds to BM-associated bacteria, and significantly lower percentages of the microbiota are coated by sIgM and sIgD, similar to those in the gut, skin, and gills of trout (4, 5, 10, 11). So far, previous studies have demonstrated that some salivary sIgA predominantly coating commensal bacteria, such as *Streptococcus mutans*, *Actinobacillus actinomycetemcomitans*, and *Porphyromonas gingivalis*, are associated with mammalian buccal diseases (99, 100). However, the type of buccal microbiota species coated by IgT/IgZ, IgM, and IgD is still unknown, which is critical to gain insights into the role of Igs in the homeostasis of the BM in teleosts.

### Immunoglobulin Responses to Microbiota in PC

The PM, as well as the ML covering the teleost PC, contains D-MALT with abundant symbiotic bacteria, but without the tonsils found in mammals (6, 101). Microbial community compositions were analyzed *via* 16S rRNA sequencing, and it was found that *Proteobacteria* and *Actinobacteria* were dominant in the pharynx of naive fish (95). However, although many studies have demonstrated the role of pharyngeal sIgA in pathogen elimination in vertebrates, limited studies have elucidated the interaction between mucosal Igs and PM microbiota. Our previous study on rainbow trout showed that pharyngeal Igs occur in response to commensal bacteria in PC, demonstrating that pharyngeal IgT coated a large population of bacteria while IgM and IgD do so to a lesser degree. Moreover, the study showed that sIgT is generated by IgT^+^ B-cells located in the PM, transferred by pIgR secreted by pIgR-expressing epithelium cells onto the pharyngeal surface, and finally binds to the microbiota (6). This fascinating similarity of IgT microbiota targeting in different MALTs may corroborate the notion that teleost MALTs form an interactive network structure and communicate with each other (4, 5, 10, 11).

## Immunoglobulin Responses Following Mucosal Immunization

Compared to systemic immunization, mucosal immunization efficiently induces a local mucosal immune response (102), which can induce “frontline immunity” with local Igs production in the MALT, neutralizing pathogens and finally preventing infection (103). Additionally, mucosal vaccines are superior to systemic vaccines from a production and regulatory standpoint (104). Mucosal immunization is simple and suitable for farmed fish immunization. Additionally, immunization *via* injection inflicts minor trauma on the body through which pathogens may easily break through and induce an inflammatory response. In contrast, mucosal immunization does not have this potential drawback. Therefore, understanding the Igs response in teleosts following mucosal immunization will be helpful for the design and development of novel fish vaccines.

### Immunoglobulin Responses Following Oral Immunization

In mammals, sIgA plays an important role in humoral immunity, which participates in adaptive immune responses to inhibit oral pathogens (80); recently, the BM of fish was proven to be a type of MALT (5). However, compared to oral immunization in mammals, oral vaccination methods in fish are still in development. Many new attempts to develop new fish immunization strategies such as the use of pathogen-coding DNA and pathogen recombinant proteins have established the basis for the potential development of oral immunization in the future (105–107). After orally treating trout with an alginate-encapsulated antigen (i.e., oral vaccination), increased numbers of IgT^+^ and IgM^+^ B-cell were observed in the pyloric caeca, which correlated with an increased expression of IgT and IgM in the same area (108). Later on, Iván et al. demonstrated that high levels of specific IgM antibodies could be detected in fish serum when oral immunizations were administered (109). Importantly, it has been proved that recombinant tumor necrosis factor a (rTNFa) could significantly improve a commercial sea bass oral vaccine against *V. anguillarum*. And during the response, gut IgT transcripts were detected to increase (110). Overall, future studies should characterize Ig responses following oral immunization in more detail.

### Immunoglobulin Responses Following Immersion Immunization

Immersion immunization is considered to be the simplest and most practical method of vaccination in aquaculture. Amend and Fender first described the method of immersion immunization in small fish, and they found that when the fish were exposed to a bovine serum albumin (BSA) solution with hyperosmotic treatment, the BSA would penetrate the juvenile trout blood, highlighting the effectiveness of immersion immunization (111). Although abundant studies on immersion immunization have been reported in different fish species, the involvement of Ig responses is relatively limited. In one study, after the fish were immersion-vaccinated with a Danish strain of *Y. ruckeri* serotype O1, biotype 2, the concentration of *Y. ruckeri* -specific IgM hardly changed compared to that of unvaccinated fish. However, after 3 weeks of exposure to live bacteria, the antibody level in vaccinated fish increased significantly (112). Similarly, another study also detected significantly increased IgM antibody levels in vaccinated rainbow trout challenged with 1 × 10^9^ CFU mL^−1^ *Y. ruckeri* bacterin for 1 h (113). Moreover, the titers of bacteria-specific antibody in rainbow trout increased when immersion vaccinated with a 2.0 × 10^8^ CFU mL^−1^ live *F. psychrophilum* suspension (114). These results suggest that immersion immunization can induce the adaptive immune response to protect the body. Additionally, upregulated mRNA expression of IgM and pIgR could be detected in vaccinated fish after *V. anguillarum* bacterin exposure and the pIgR expression levels were higher in some mucosal tissues (gills, skin, and hindgut) (115). Importantly, the delivery of live attenuated vaccines can improve the immune ability of fish (116). For instance, after fish fry were bath vaccinated with a live *F. columnare* vaccine, the survival rate of channel catfish (*Ictalurus punctatus*) increased between 57% and 94% (117). Moreover, another study reported that zebrafish vaccinated with live attenuated *Vibrio anguillarum* possessed more strong mucosal immune responses (118). However, despite these observations, further studies on teleost IgT/IgZ response and IgM and IgD response in fish lacking IgT following immersion immunization are still needed.

### Immunoglobulin Responses Following Anal Immunization

The presentation of M-like cells in the intestine suggest that antigens could be uptaken (119), thereby inducing strong immunity in the infected or vaccinated fish. A recent study showed that, compared with oral vaccination, single-dose anal bacterin intubation following bath challenge with *Y. ruckeri* rendered a high survival rate in teleost fish, suggesting that immunizing antigens might have been destroyed in the stomach of the oral vaccination group. However, in the same study, no significant differences in the IgM antibody levels were detected in the plasma of the two vaccination groups at any of the sampling time points (120). A later study also demonstrated that there were no significant differences in IgM levels of anally-intubated fish, yet the expression of sIgD and IgT were upregulated in both the intestines of fish immunized *via* anal intubation and in the gills of fish immunized by immersion (121). Recently, the anal administration of thymus-independent (TI) antigens without additional adjuvants was investigated in rainbow trout. An efficient B-cell response was induced, which facilitated the production of specific IgM in serum and the differentiation of antigen-specific ASCs (122).

### Immunoglobulin Responses Following Nasal Immunization

Compared with other vaccination types, nasal vaccination, which is an accessible method to control infectious diseases (123), has the advantages of using a needle-free delivery system and a lower dosage of antigen to elicit an immune response. Nasal vaccines are widely used in many animals such as cats, dogs, and cattle. Nasal vaccination in mammals induces IgA-specific responses in O-NALT and D-NALT (124). Recently, the discovery of NALT in rainbow trout prompted studies concerning nasal vaccines for use in aquaculture (12). Thereafter, it was found that nasal vaccination can effectively protect fish against viral and bacterial pathogens (125). Furthermore, the nasal vaccinated group with live attenuated IHNV could significantly reduce mortalities of the trout after the IHNV infection, whereas nasal vaccinated group with formalin killed *Y. ruckeri* appear to fully protect fish against enteric red mouth (ERM) disease (126). Moreover, nasal vaccination with ERM bacterin also generated different IgM and IgT repertoire dynamics at systemic and mucosal levels and induced striking IgT responses in the spleen of rainbow trout (78).

## Concluding Remarks

This review sought to gather recent advances that have shed some light on the immune responses of Igs in teleost fish to microorganisms or immunization, including mucosal pathogens, vaccines and microbiota. Teleost sIgM, sIgT, and sIgD coexist at all mucosal sites, including the gut, gills, olfactory organ, skin, BC, and PC. During pathogenic infection, specific sIgT can be locally produced in mucosal secretions, and mucosal sensitivity increases in IgT-depleted fish, while large quantities of specific sIgM are generated in the serum. These findings suggest that the IgT-mediated immune response is crucial in all mucosal sites, whereas IgM responses are typically systemic in all teleosts and also functional in mucosal sites of teleosts which lack IgT/Z (7, 77). Considering that IgM has the highest protein concentration among teleost Igs on mucosal surfaces and that the specific IgM also can be induced in the mucosal surfaces by parasites as in the serum, it is worth investigating whether sIgM has an auxiliary effect on sIgT to eliminate pathogens at mucosal sites. Additionally, mucosal responses of Igs in fish, mainly trout, have been demonstrated after parasitic and bacterial infection (4, 5, 10, 11, 74, 77), but fish mucosal responses after infection by other pathogens such as virus or fungi are still unclear. In vertebrates, the common mucosal immune system (CMIS), which means that antigens administration induces humoral immune responses not only at the mucosal site of antigen application but also in other external mucosal tissues due to the dissemination of antigen-sensitized cells, is vitally important. Specifically, mammalian has been found to produce specific IgA or IgM responses in other MALTs upon stimulation of one mucosal site (127); however, data on the CMIS in teleosts remains currently limited. Therefore, our current understanding of the mechanisms underlying teleost Ig responses at mucosal sites remains nascent. Especially, the humoral immune responses in teleosts which lack IgT/Z remain much unknown. Future studies need to consider the role of IgT, IgM, and IgD in mucosal immunity to different pathogens by infection or immunization and explore the CMIS in teleosts, which will contribute to the development of fish vaccines.

Another very important point is that the mucosal microbiota is predominantly coated by teleost IgT at mucosal sites. IgT depletion induces a profound dysbiosis, the expansion of pathobionts, tissue damage, the translocation of mucosal bacteria, and inflammation. These findings demonstrate the previously unrecognized ability of IgT in immune exclusion to protect the mucosa from microbiota (7). However, information on the role of teleost sIgT in bacterial colonization remains scarce. Understanding how sIgT coating of bacteria mediates bacterial colonization under homeostatic conditions may improve the efficiency of aquatic probiotics. Moreover, it is worth noting that teleost IgM can bind a subset of microbiota, especially in the teleost olfactory organ, and the percentage of IgM-coating bacteria in fish is notably higher than that in mammals (10, 12, 96). Importantly, in fish devoid of IgT, the percentage of microbiota coated by IgM is significantly higher, suggesting that compensatory sIgM might then coat microbiota that was originally coated by sIgT (7). Thus, the function of sIgM in teleost commensal homeostasis has not yet been determined, and future studies based on IgM depletion experiments may provide important insights into this matter. Additionally, mammalian IgA-derived antibodies are commonly polyreactive and show low-affinity to numerous microbial antigens. Although teleost mucosal Ig (IgT) has been found to target a taxonomically distinct subset of microbiota at the gill surface (7), the extent of the microbe recognition capacity of IgT as well as its mucosal-specific pathogen-binding capacity during infection are still unknown.

Overall, it seems clear that Igs in teleosts, similar to those of mammals, can defend mucosa against pathogens for elimination and interact with microbiota to maintain commensal homeostasis; however, many aspects of their responses remain poorly understood. Thus, the inner mechanisms of Igs regulating both the pathogenic clearance and commensal homeostasis should be determined in the future research.

## Author Contributions

All authors contributed to the article and approved the submitted version.

## Funding

This work was supported by grants from the National Natural Science Foundation of China (U1905204, 31873045) and the Key Laboratory of Marine Biotechnology of Fujian Province (2020MB03).

## Conflict of Interest

The authors declare that the research was conducted in the absence of any commercial or financial relationships that could be construed as a potential conflict of interest.
